# Sporadic Cerebro-Spinal Meningitis

**Published:** 1901-06-08

**Authors:** 


### SPORADIC CEREBRO-SPINAL MENINGITIS.

THERE has long been a question as to the exact position and relationship of the disease known as cerebro-spinal meningitis. It was admitted by most observers that a diplococcus was invariably found in the meninges in cases of meningitis, but considerable differences existed as to its precise nature: some regarded it as the pneumococcus of Fraenkel, or as a variety of this organism; while others looked upon it as a specific microbe. It has, of course, to be admitted that the pneumococcus may produce a meningitis, or may be present as a secondary infection in a meningitis arising from some other cause; indeed, it is generally recognised that meningitis often complicates pneumonia, and *vice versâ*. But to say that the pneumococcus is the specific agent at work in the production of the disease cerebro-meningitis, as we know it in its epidemic and sporadic form, is another matter. In view of more recent investigations there is much reason for believing that the diplococcus of Weichselbaum is the real cause of cerebro-spinal meningitis, and that this organism is something quite distinct from the pneumococcus. A very interesting point in regard to the relationships of this disease is the observation made a couple of years ago by Dr. Still in regard to the disease which is known as posterior basal meningitis of children. This he was inclined to regard as merely a sporadic manifestation of cerebro-spinal meningitis, and in support of this view he gave the results of the bacteriological examination of a number of cases in which he invariably found intra- and extra-cellular diplococci which morphologically resembled those described by Weichselbaum. It evidently would simplify matters very much if we could regard both epidemic and sporadic cerebro-meningitis and the posterior basal meningitis of children as all being manifestations of the invasion of the same micro-organism. Lately Dr. William Hunter and Mr. Alexander Nuthall,[1] of the London Hospital, have had the opportunity of examining bacteriologically the cerebro-spinal fluid obtained by lumbar puncture from nine cases of meningitis in children and from one case in a young adult. It was difficult from a clinical point of view to determine the exact nature of these cases of meningitis—in fact, it was impossible to say with any degree of certainty whether they were simple or tuberculous. On this account lumbar puncture was performed, and the results of the bacteriological examination of the cerebro-spinal fluid thus obtained proved of great value in the determination of the exact nature of the morbid processes present. The cases are given in detail, and the authors conclude as follows:—"1. That in all the cases of meningitis examined by us a diplococcus has been isolated from the cerebro-spinal fluid. In nine of these the fluid was obtained by lumbar puncture during life. 2. That this diplococcus has the same morphological and biological characteristics as Weichselbaum's diplococcus intracellularis meningitides. 3. That in some cases the diplococcus was present in pure culture, in others associated with other micro-organisms—e.g. bacillus influenzæ and bacillus tuberculosis. 4. That the clinical picture and the pathological changes found in these cases are those met with in so-called 'posterior basal meningitis.' 5. That in all probability 'posterior basal meningitis' is simply a sporadic manifestation of cerebro-spinal meningitis, and is produced by the same micro-organism, namely, the diplococcus intracellularis meningitidis."

[1] Lancet, June 1.

